# Advancing regional and international cooperation in public health diplomacy: summary of the methods and outcomes of the 2nd University of Memphis School of Public Health Diplomacy Summit (2026)

**DOI:** 10.3389/fpubh.2026.1923425

**Published:** 2026-09-04

**Authors:** Ashish Joshi, Ashraf El-Metwally, Luis de Almeida Sampaio, Antonio Sarria-Santamera, Erica Kastrup, Ramune Kalediene, Josep Figueras, Robert Otok, Woldekidan Amde, Rajendra Surenthirakumaran, Rodrigo Reis, William Yotive, Matthew Brown, Brian B. McGoldrick, Niharika Jha, Kun-Hsien Tsai, Xinhua Yu, Marian Levy, Maryam Karimi, Paul Barach, Gretchen Peterson, Diana Ruggiero, Dorothy Biberman, Tiruneh Baye, Beverly Tsacoyianis, Shawna Novak, Kamiar Alaei, Ntuli A. Kapologwe, John Middleton, Henrique Barros, Margaret Kaseje, Olagoke Akintola, Wah Yun Low, Michael Arthur Ofori, Sharon Denise Griffin, Nishtha Sharma, Savannah Candace Kilgore, Kami Geron, Lucia Linares, Shaun Palmer, Olha-Mariia Alimenko, Diana Maddah, Laura Magaña

**Affiliations:** 1 School of Public Health, The University of Memphis, Memphis, TN, United States; 2 King Saud bin Abdulaziz University for Health Sciences, Riyadh, Saudi Arabia; 3 Coimbra Health School, Coimbra, Portugal; 4 School of Medicine, Nazarbayev University, Astana, Kazakhstan; 5 Center for International Relations in Health (CRIS), Oswaldo Cruz Foundation, Rio de Janeiro, Brazil; 6 Department of Health Management, Lithuanian University of Health Sciences, Kaunas, Lithuania; 7 European Observatory on Health Systems and Policies, London, United Kingdom; 8 Association of Schools of Public Health in the European Region (ASPHER), Brussels, Belgium; 9 School of Public Health, University of the Western Cape, Cape Town, South Africa; 10 Department of Community and Family Medicine, University of Jaffna, Jaffna, Sri Lanka; 11 Bursky School of Public Health, Washington University in St. Louis, St. Louis, MO, United States; 12 World Federation of United Nations Associations (WFUNA), New York, NY, United States; 13 School of Diplomacy and International Relations, Seton Hall University, NJ, United States; 14 Department of Public Health, National Taiwan University, Taipei, Taiwan; 15 Jefferson College of Population Health, Thomas Jefferson University, Philadelphia, PA, United States; 16 Department of Sociology, University of Memphis, Memphis, TN, United States; 17 Department of World Languages and Literatures, University of Memphis, Memphis, TN, United States; 18 Association of Schools and Programs of Public Health (ASPPH), Washington, DC, United States; 19 Africa Centres for Disease Control and Prevention (Africa CDC), Addis Ababa, Ethiopia; 20 Department of History, University of Memphis, Memphis, TN, United States; 21 Canada International Scientific Exchange Program (CISEPO), Toronto, ON, Canada; 22 Department of Health Science, California State University, Long Beach, CA, United States; 23 Association of Schools of Public Health in Africa (ASPHA), Dar es Salaam, Tanzania; 24 Global Network for Academic Public Health (GNAPH), Washington, DC, United States; 25 Department of Clinical Epidemiology, University of Porto, Porto, Portugal; 26 Association of Schools of Public Health in Africa (ASPHA), Nairobi, Kenya; 27 School of Public Health, University of the Western Cape, Cape Town, South Africa; 28 Department of Social and Preventive Medicine, Universitiy Malaya, Kuala Lumpur, Malaysia; 29 Young Forum Gastein, Bad Hofgastein, Austria; 30 Young Forum Gastein, Amsterdam, Netherlands; 31 Young Forum Gastein, Kyiv City, Ukraine; 32 UK-Med, Doha, Qatar

**Keywords:** diplomacy competencies, global health governance, health diplomacy education, international cooperation, public health diplomacy, thematic analysis, workforce development

## Abstract

Public health diplomacy has emerged as a critical mechanism for addressing complex global health challenges. This paper summarizes the key discussions, lessons learned, and recommendations from the Public Health Diplomacy 2nd Summit, organized by University of Memphis. In the summit, participants explored the role of public health diplomacy in strengthening health systems and building resilient partnerships. This paper employed a qualitative descriptive approach for the data analysis. Participants emphasized the need to integrate diplomacy competencies into public health education and professional training while fostering stronger connections between evidence generation and policy action. The findings suggest that public health diplomacy should be recognized as both a strategic and operational tool for strengthening global health governance, promoting international cooperation, and preparing future public health leaders.

## Introduction

Public health challenges in the 21st century, including pandemics, climate change, humanitarian crises, and widening health quality inequities, require coordinated, cross-sectoral, and transnational responses. Public health diplomacy has emerged as a multidisciplinary field that integrates public health, foreign policy, development, human rights and global security to address these challenges through collaboration, negotiation, and consensus-building. It promotes engagement among governments, academia, civil society, and international organizations to advance global health quality, equity and collective well-being (1). In recent years, the importance of public health diplomacy has become increasingly evident in the global arena, particularly in response to complex and transboundary health challenges. Events such as the COVID-19 pandemic underscored the critical need for robust international cooperation, transparent and trusting communication, and coordinated policy responses to manage public health emergencies (2). At the same time, ongoing issues related to climate change, gender equality, migration, conflict, and resource inequities have further highlighted the interconnected nature of health and foreign policy (3, 4). Public health diplomacy serves as a strategic mechanism to bridge gaps between sectors and nations, foster trust among stakeholders, and facilitate collective action to address shared health priorities (4, 5).

The University of Memphis School of Public Health has played a pioneering role in institutionalizing this effort by establishing the nation’s first Public Health Diplomacy Lab (6). The Lab was created to bring together diverse stakeholders, including governments, non-governmental organizations, academic institutions, and civil society, to strengthen collaboration and coordination for improving health outcomes through diplomacy-driven approaches. Building on this foundation, the inaugural Public Health Diplomacy Summit held in 2024 resulted in a policy brief, which defined public health diplomacy and proposed a global action agenda (1). The summit utilized a human-centered, participatory framework and produced key outputs, including a working definition emphasizing interdisciplinary collaboration, communication, negotiation, and consensus-building, as well as a multi-point action plan to institutionalize the field and build global capacity (7).

Expanding on these efforts, the 2nd Public Health Diplomacy Summit was held from March 23–25, 2026, under the theme of *“Regional and International Cooperation to Advance Public Health Diplomacy among Schools and Programs of Public Health.”* The summit convened a diverse group of stakeholders, including academicians, diplomats, civil society members, and global partners from 16 countries, to further advance the field and strengthen international collaboration (8). A key objective of the 2026 summit was to move beyond conceptualization toward operationalization by identifying competencies, skills, and strategies required for public health diplomacy practice. These included leadership, negotiation, communication, and consensus-building in the context of increasingly complex and uncertain global health environments.

Central to the summit were structured discussions, where participants engaged with guiding questions aimed at strengthening public health diplomacy education, fostering global partnerships, and translating diplomatic principles into actionable public health interventions. These discussions generated a set of collective outcomes and recommendations focused on advancing workforce capacity, enhancing regional and international collaboration, and embedding public health diplomacy within academic and practice settings. This paper builds on the methodological foundation and findings of the 2024 summit and aims to synthesize the outcomes of the 2026 Public Health Diplomacy Summit.

## Methodology

### Study design

This study adopted a qualitative, participatory design to analyze and synthesize the outcomes of the 2026 Public Health Diplomacy Summit. The approach is grounded in the human-centered design (HCD) framework and collaborative methodology used in the 2024 summit, as documented in our earlier publication (1). This framework emphasizes iterative engagement, stakeholder participation, and consensus-building to generate actionable insights and recommendations (9).

### Study setting and participants

**Figure 1:**
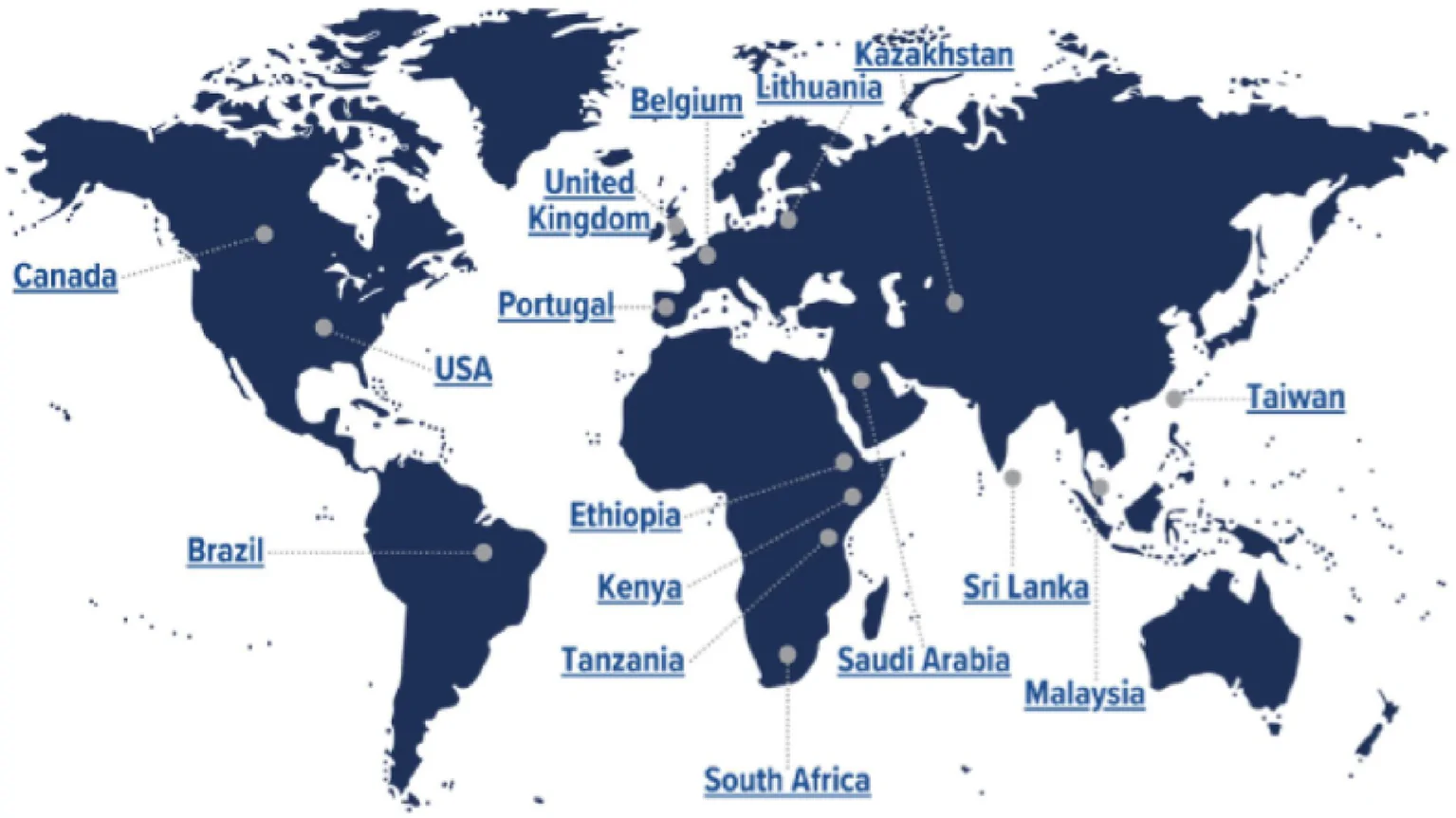
Map of member countries of origin.

The 2026 summit was held from March 23–25 at the University of Memphis and brought together a diverse group of stakeholders in person, including academicians, public health professionals, diplomats, civil society representatives, and partner organizations. Participants represented 16 countries, 7 regions, and 11 public health organizations ensuring a global and interdisciplinary perspective on public health diplomacy (Figure 1, Table 1).

**Table 1:** Public health/academic organizations (represented at the 2nd Public Health Diplomacy Summit).

Academic institution	Institution location	Organization	Organization geographical classification
California State University, Long Beach	United States of America	Africa Centers for Disease Control (ACDC)	Regional
King Saud bin Abdulaziz University for Health Sciences	Saudi Arabia	Asia Pacific Academic Consortium for Public Health (APACPH)	Regional
Lithuanian University of Health Sciences	Lithuania	Agency for Public Health Education Accreditation (APHEA)	Global
National Taiwan University	Taiwan	Alianza Latinoamericana de Salud Global (Latin American Alliance for Global Health) (ALASAG)	Regional
School of Medicine of Nazarbayev University	Kazakhstan	Association of Schools of Public Health in the European Region (ASPHER)	Regional
Seton Hall University, School of Diplomacy and International Relations	United States of America	Association of Schools and Programs of Public Health (ASPPH)	Regional
Thomas Jefferson University	United States of America	Canada International Scientific Exchange Program (CISEPO)	Global
Universti Malaya	Malaysia	Coimbra Health	Regional
University of Jaffna	Sri Lanka	CRIS Center for International Relations in Health of the Oswaldo Cruz Foundation (CRIS FIOCRUZ)	Global
University of Memphis	United States of America	East, Central, and Southern Africa Health Community (ECSA-SH)	Regional
University of Porto	Portugal	European Observatory on Health Systems and Policies	Regional
University of the Western Cape School of Public Health	South Africa	Global Network for Academic Public Health (GNAPH)	Global
Washington University in St. Louis	United States of America	South-East Asia Public Health Education Institution Network (SEAPHEIN)	Regional
Lithuanian University of Health Sciences	Lithuania	UK – MED	Regional
		World Federation of United Nations Association (WFUNA)	Global
		World Health Summit (WHS)	Global

### Data collection

Data was collected from structured panel sessions (*n* = 6) and facilitated discussions (*n* = 4) conducted during the summit. According to the summit program, Day one focused on welcoming the participants, and organizing the networking session. Day 2 consisted of five sessions, each designed to capture regional perspectives and advance dialogue on public health diplomacy (8).

Each session featured 3–4 panelists representing different countries or regions. Panelists presented the status, progress, and ongoing initiatives related to public health diplomacy within their respective contexts, highlighting regional priorities, challenges, and opportunities for collaboration. These presentations provided foundational input for subsequent discussions.

Following the panel presentations, each session included a moderated discussion segment in which participants engaged collectively to reflect on key issues, share experiences, and identify common priorities. All sessions were guided by a set of pre-structured questions (Table 2), developed to ensure consistency across sessions and to elicit insights on core domains of public health diplomacy, including education and training, regional and global collaboration, workforce development, and the translation of diplomacy into public health practice.

**Table 2:** Pre-structured questions for each session.

Sessions	Topic	Pre-structured questions	Description
1	A Paradigm Shift from Global Health Diplomacy to Public Health Diplomacy	How does public health diplomacy require a blend of technical public health expertise and diplomatic skills to navigate the intersection of health, foreign policy, and economic interests?	The shift from global health diplomacy to public health diplomacy marks a transition from top-down initiatives to collaborative, sustainable strategies that treat health as a key component of foreign policy and security. This development prioritizes local capacity building, mutually beneficial partnerships, and multi-stakeholder engagement to address transnational health threats while strengthening local and national health systems.
2	Public Health Diplomacy: Lessons Learned and Opportunities Ahead	What are some of the lessons and opportunities toward implementation of public health diplomacy initiatives across diverse local, national and global settings?	Public Health Diplomacy lessons emphasize the need for trusted networks, evidence-based policy, and navigating diverse institutional contexts along with strengthening international cooperation, while navigating legal, financial, and cultural barriers.
3	Health Security and Public Health Diplomacy in an African Context	Discuss strategies that could utilize public health diplomacy as a tool to advance health security, and good health and well-being in Africa and beyond.	Health security and public health diplomacy in Africa focus on regional cooperation, strengthening health systems, and achieving health sovereignty to address pandemic preparedness, disease surveillance, universal health care and financing of healthcare delivery services.
4	Advancing Public Health Diplomacy in Europe: ASPHER Perspective	How must public health diplomacy evolve into a bold, inclusive, and strategic force to defend evidence, empower diverse actors, and reform institutions so that it can safeguard health as a foundation for peace and global progress?	Public health is under pressure from rising populism, disinformation, and weakened global institutions, threatening cooperation, equity, and trust in science.
5	Engaging Public Health Students and Practitioners in Applied Public Health Diplomacy Initiatives	What are some ways to engage public health students and practitioners in applied public health diplomacy initiatives toward creating a public health diplomacy workforce?	Engaging public health students and practitioners in applied public health diplomacy involves bridging the gap between academic theory and real-world negotiation, focusing on skills like crisis management, cultural sensitivity, and multi-stakeholder collaboration.

Facilitators and note takers documented the discussions across sessions, capturing key insights, recurring themes, consensus points, and proposed recommendations. Additional data sources included summit materials such as the official agenda, session summaries, and compiled notes from each session.

### Data analysis

A thematic analysis approach was employed to characterize key themes and takeaways from the qualitative data generated from the panel discussions and session dialogues. Notes, summaries, and transcriptions from each session were systematically reviewed and coded to identify recurring themes, patterns, and priority areas emerging across different regions and stakeholder perspectives. Coding is the interpretative process in which conceptual labels are given to the data (10).

A positivist scope was applied to capture and analyze the sentiments of each speaker and conversation with as little interpretation as possible from the notetakers or analyst, while acknowledging that some bias is inevitable (11). We used an inductive process of moving from individual observations and points to form broader themes that characterized the discussions and summit holistically. First, initial descriptive coding was conducted by two independent and trained coders to capture key concepts and ideas reflected in the discussion notes. These codes were then grouped via thematic coding into subthemes representing common areas of focus across sessions. In the final stage, broader themes were developed and then refined through comparison across all sessions to ensure consistency, coherence, and representativeness of the collective perspectives shared during the summit. Any discrepancies during coding were solved through consensus.

The analysis was specifically chosen to identify major thematic areas emerging from the discussions, synthesize consensus-based outcomes, and generate actionable recommendations. These findings were intended to inform strategies for advancing public health diplomacy at both regional and global levels, with particular emphasis in understanding how PHD adapts to varied regional geopolitical architectures and strengthening collaboration, capacity building, and practical implementation. MAXQDA 24 was used for the organization, coding, and analysis of documents (12).

### Ethical considerations

This study is based on aggregated discussions conducted in a professional summit setting and does not involve individual-level identifiable data. Formal Ethical approval was not required.

## Results

A thematic analysis was conducted on a corpus of notes and transcripts from the second day of a 3-day international public health diplomacy summit. Following an analysis of the notes and triangulation with the recorded transcripts, a total of 91 codes were initially created. After review, 6 themes (Collaboration in Public Health Diplomacy, Facilitation and Implementation of Initiatives, Professionalization of Public Health Diplomacy, Social Political, and Economic Context of Public Health Diplomacy, Conceptual Development of Public Health Diplomacy, and Evaluation of Initiatives and Translation of Findings) were developed with a group of 23 subthemes. A definition of each larger theme can be found in Table 3, while the full collection of themes, subthemes, and their definitions can be found in Table 4.

**Table 3:** Themes and definitions.

Theme	Definition
1. Collaboration in Public Health Diplomacy	Initiatives require interdisciplinary, culturally competent, and systematic collaboration.
2. Facilitation and Implementation of Initiatives	Comprehensive system strengthening is needed to develop and advance the field.
3. Professionalization of Public Health Diplomacy	The field needs to move toward professionalization and academic development.
4. Social, Political, and Economic Context of Public Health Diplomacy	The field should strive for outcomes that consider various idiosyncrasies and contexts.
5. Conceptual Development of Public Health Diplomacy	To move the field forward, stakeholders should strive for definitional clarity and research priorities.
6. Evaluation of Initiatives and Translation of Findings	A comprehensive evaluation framework and translation protocol should be established for initiatives.

**Table 4:** Themes, subthemes, definitions, and representative quotations.

Theme	Subtheme	Definition	Representative textual data
Collaboration in Public Health Diplomacy	Strengthening Communication Networks	Develop and maintain communication channels between actors and organizations	“One of which [that] was highlighted is the need to strengthen and tailor communications to foster dialogue across diverse, and at times, opposing stakeholders”
	Interdisciplinary Collaboration	Interdisciplinary collaboration is crucial to implementing public health diplomacy initiatives.	“…We need to advance more in [sic] interprofessional and cross-cutting public health.”
	Bridging Cultural Perspectives	Public Health Diplomacy requires the acknowledgement and integration of cultural perspectives and idiosyncrasies	“Multidisciplinary skills are required, including language, communication skills, and the ability to breakdown cultural barriers…”
	Bridging Academic and Non-Academic Perspectives	Funding The author(s) declare that no financial support was received for the research and/or publication of this article. Conflict of Interest Statement The authors declare that the research was conducted in the absence of any commercial or financial relationships that could be construed as a potential conflict of interest. Acknowledging and integrating extra-academic viewpoints	“As academics in this [we have] to make sure that we have 1 foot academia but also one foot in policy in this environment”
Facilitation and Implementation of Initiatives	Adopting Implementation Science into Public Health Diplomacy	Implementation frameworks are necessary to effectively translate evidence into working policy	“…The improvement science, implementation science, and the barrier-addressing sciences – we have 50 year of knowledge [on] why great ideas fall short”
	Financial System Strengthening and Innovation	Public health diplomacy development and facilitation necessitate financial support outside of traditional research funding avenues.	“Do not be afraid of the private sector, because those innovative financial methods can be very valuable and there’s interest.”
	Technological System Strengthening	Embracing novel technology can help address otherwise unnavigable diplomatic and collaborative challenges.	“I think languages are so important. That’s not something for us to neglect in any way. And these days, with the advents of new technologies and agents, agentic AI, there’s no reason to not be inclusive”
Professionalization of Public Health Diplomacy	Developing Competency Frameworks	A standardized competency framework can help clarify training needs for budding public health diplomats.	“…We also need rigorous development of competency models at the multi-stakeholder and the informal [level]…I challenge you to be more rigorous with our definitional clarity so that we have the aligned actors with aligned competency models”
	Assessing PHD Workforce Needs	Requisite needs and skill for the public health diplomacy workforce need to be identified.	“…the kinds of competencies that public health needs to have, leadership, systems thinking, communication and so on”
	Developing PHD curriculum	Discussion regarding the development and implementation of an academic curriculum in public health diplomacy.	“We are working to organize education and modernize the curriculum. The goal is clear: to ensure that professionals are not only technically competent, but also capable of navigating policy, governance, and international cooperation.”
	Credentialing	Discussion regarding the utility of credentialing or identifying PHD professionals	“To also give the recognition of public health graduates and professionals with the strong component of public health diplomacy…through [sic] those technical documents such as competency frameworks, core curriculums, accreditation standards, and professional credentials”
	Bridging PHD and Extant PH Field	Public health diplomacy needs to be recognized and integrated into the broader field of public health.	“Can we glom onto and strengthen existing networks of public health schools that have practical implications?”
Social, Political, and Economic Context of Public Health Diplomacy	Impact of Political Climates on Public Health Diplomacy	Discussion of the impact of political climates (macro or otherwise) on PHD and PH broadly.	“Health has changed…It is now strategic. It is now political. It is now global. The pandemics, the climate change, the inequalities. Health and disease ignore borders. “
	Role of Public Health Diplomacy in Conflict	Discussion of PHD’s utility and role in addressing public health needs in conflict (macro or otherwise)	“We saw [health diplomacy] as a tool for collaborating in spite of the conflict that was going on. As you can see the map, the political divide was there…Something had to be done because health was at stake. Health security was being compromised.”
	Responses to Public Health Crises	Public health diplomacy can uniquely contribute to public health crisis response.	“That was one of the failures of COVID; public health professionals not being able to target their messages to the political leaders in a timely way”
	Cultural competency in public health diplomacy	Cultural competency is a key skill for effective public health diplomacy action.	“Solutions are developed under other realities practicing cross-cultural listening”
	People-Centered Outcomes	Public health diplomatic efforts need to strive for people-centered outcomes that consider the cultural and pragmatic needs of the target group.	“Working with local people, a series of actions were carried out, such as the vector control. Nationwide malaria screening, household visits, rapid tests, administration of ACT prophylactic medication. The results were remarkable.”
Conceptual Development of Public Health Diplomacy	Defining Public Health Diplomacy	Discussion regarding the need or lack thereof a universal definition for PHD	“About these definitional terms. So the way that we go about looking at global health diplomacy versus public health diplomacy and trying to get at the crux of how to effectively situate it on a ‘glocal’ basis”
	Characterizing regional priorities	Discussion regarding how distinct geopolitical and regional architectures shape localized PHD priorities and frameworks	“Perhaps what we call “local” is too much of a simplification because the regional groupings play such a large role in addressing public health – and so I think we need to think of it more in terms of local, regional, and global…”
	Knowledge/Resource Creation	Discussion regarding the importance of developing a dynamic knowledge/resource base.	“…Support public health diplomacy labs and other training environments…. Living repositories of briefs, case studies, and best practices.”
	Development of research base	Discussion regarding the facilitation and importance of PHD research.	“We have to make sure that whatever we do has policy and social dimensions and implications, but at the same time has to be at the highest level of research and evidence…”
Evaluation of Initiatives in Public Health Diplomacy	Latent Outcomes of Public Health Diplomacy	Latent outcomes need just as much urgency and consideration as salient health outcomes (Trust, Resilience, etc.)	“Public health diplomacy is enabling directives. It’s about listening…if you do that, you [sic] get more trust and you are more sustainable”
	Salient Outcomes of Public Health Diplomacy	Discussion and characterization of salient outcomes of PHD initiatives (disease mitigation, increased health behaviors, etc.)	“After 10 years of hard work, the hospital beds have finally become available and the mosquito nets can be rolled up”
	Development of an evaluation framework	Development of a standardized evaluation framework that can be used to assess the efficacy and impact of public health diplomacy efforts is crucial.	“…if we are if we are looking at this as being something of a barrier to us doing practical, pragmatic work that’s very needs-specific and that also is that we are able to incorporate proper monitoring and evaluation, proper measurement that uses implementation science methodology and mixed methods, not just the quantitative, but the qualitative to characterize it…”

### Themes from summit sessions

Our findings indicate the vital importance of both applied and developing conceptual and operational aspects of Public Health Diplomacy. Many of the topics reflect continued development and thought about topics raised at the first public health diplomacy summit, while others indicate novel discoveries from the attendees’ respective perspectives and lived experiences (1).

Collaboration was a cross-cutting theme across all presentations and discussions, particularly regarding interdisciplinary collaboration (i.e., collaborating between public health and non-public professionals) and bridging traditionally siloed perspectives (i.e., cultural differences, academic vs. non-academic) to build trust. Furthermore, there was a call for increased communication between relevant parties (‘strengthening communication networks’) that would facilitate collaborative initiatives and the sharing of operational insights.

*“We need more resilient partnerships in core, multi-stakeholder and informal [partnerships], and we need to engage more partners in global health action.”*

Discussions relevant to the theme of ‘Facilitation and Implementation of Initiatives’ addressed streamlining the mechanisms behind public health diplomacy. The integration of implementation science in public health diplomacy was suggested. As structured institutional and organizational collaborations are developed, attendees suggested standards for translating evidence to policy are necessary to effectively realize public health diplomacy efforts. In addition to implementation frameworks, innovation in technology use and financial system strengthening were emphasized across the summit. Attendees suggested engaging with private organizations, businesses, and other potential stakeholders as a funding strategy, particularly in the face of diminished traditional public health funding. Furthermore, attendees discussed the need to embrace technological innovation to optimize certain public health diplomacy processes (i.e., using agentic AI in the context of implementation workflows, generative AI to facilitate language translation, health technology for assessment of public health diplomacy outcomes, etc.).

*“… one of the biggest things we find is understanding the gap between the big ideas and measurable system transformation… We have 50 years of knowledge, why [do] great ideas fall short?”*

*“I think languages are so important. That’s not something for us to neglect in any way. And these days, with the advents of new technologies, agents, and agentic AI, there’s no reason to not be inclusive.”*

*“…one of the ways forward that has not been mentioned yet beyond flexible funding using innovative financial mechanisms…Do not be afraid of the private sector, because those innovative financial methods can be very valuable….”*

The idea of professionalization and legitimizing public health diplomacy was heavily discussed throughout the summit, with the resultant subthemes covering professionalization in academia, the workforce, and beyond (‘Developing competency frameworks’, ‘Assessing PHD workforce needs’, ‘Developing PHD curriculum’, ‘Credentialing’, and ‘Bridging PHD and Extant PH field’). Required Public Health Diplomacy competencies were a focus of discussion at the first UM Public Health Diplomacy summit, while talks at the second summit focused predominantly on collating the competencies into a standardized framework that can be applied across multiple contexts. To support adoption and implementation, participants highlighted the need to align public health diplomacy competencies with established frameworks, such as CEPH competencies, ensuring their integration into accredited public health curricula and workforce development initiatives. Furthermore, the need to understand practical workforce needs for a budding public health diplomacy workforce. Finally, the need to fully bridge the public health diplomacy with the extant public health field was discussed in order introduce diplomacy concepts into public health education, accreditation, and research.

“*We need state to state actors, but we also need rigorous development of competency models at the multi-stakeholder and informal [levels].”*

*“To also give the recognition of public health graduates and professionals with a strong component of public health diplomacy now…through those documents such as the competency framework, the core curriculum, accreditation standards and professional credentials.”*

The theme of ‘Social, Political, and Economic Contexts in Public Health Diplomacy’ was present across multiple presentations and discussions throughout the summit. Attendees representing a variety of regions and nations acknowledged that the political climates and idiosyncrasies impact public health systems, necessitating public health diplomacy as a vehicle for resolution.

*“…how to address corruption with reform efforts being derailed by political resistance, while ensuring accountability, they need to be very diplomatic on how we go about this.”*

The theme of ‘Conceptual Development of Public Health Diplomacy’ echoes topics covered at the first Public Health Diplomacy summit. Establishing a consensus definition of public health diplomacy was one of the primary deliverables from the first summit. This conversation continued at the second summit, with attendees calling for additional nuance to the definition of public health diplomacy in different regional contexts (‘Defining Public Health Diplomacy, ‘Characterizing regional priorities’). Alternatively, other attendees maintained the need for a centralized definition that allowed for dynamic application. In addition to building on the topic of definition, the subthemes of ‘Knowledge/resource creation’ and ‘Development of a research base’ built upon discussions from the first summit regarding the creation of educational resources. Specifically, attendees called for the formation of case study repositories and research collectives to bolster the extant public health diplomacy literature and enable the sharing of best practices and methodologies.

*“And that’s an opportunity itself, the fact that we are defining this as we move forward, but not to think of these as closed doors, but [sic] passageways for us to be able to open up and link in with each other to see where are those resources.”*

*“…support public health diplomacy labs and other training environments, establishing shared evidence to policy and communication hubs, living repositories of briefs and case studies and [sic] best practices.”*

The final theme of ‘Evaluation of Initiatives in Public Health Diplomacy’ was not explicitly reflected in the proposed action items but was a ubiquitous topic throughout the discussion. As attendees presented reports and case studies of their own public health diplomacy initiatives, discussions arose regarding priority outcomes and how initiatives should be scrutinized. For example, multiple participants emphasized the importance of trust as a latent outcome; suggesting that to advance public health diplomacy professionals must look beyond health outcomes as a measure of success. However, attendees also emphasized the importance of salient health outcomes from public health diplomacy initiatives, particularly within the context of other contextual subthemes like ‘bridging cultural perspectives’ and ‘translating evidence to policy’.

“*…I said, so maybe trust here is not just a process measure, it’s actually an outcome indicator. So maybe we have to actually measure the trust.”*

*“…we are able to incorporate proper monitoring and evaluation, proper measurement that uses implementation science methodology and mixed methods, not just the quantitative, but the qualitative to characterize it….”*

To illustrate the practical relevance of the identified themes, Table 5 presents examples from the WHO Pandemic Agreement alongside potential educational applications. The WHO Pandemic Agreement exemplifies public health diplomacy by fostering international negotiation, collaboration, and consensus-building to improve global preparedness and equitable responses to future pandemics (13). This table demonstrates how the proposed public health diplomacy concepts can inform policy practice, competency development, and curriculum design within schools and programs of public health.

**Table 5:** Practical application of public health diplomacy themes using the example of WHO pandemic agreement and corresponding educational strategies.

Theme	Pandemic Agreement Example	Educational Application
1. Collaboration in Public Health Diplomacy	International treaty negotiations	Interprofessional teamwork exercises
2. Facilitation and Implementation of Initiatives	Global surveillance systems	Health policy implementation projects
3. Professionalization of Public Health Diplomacy	Global workforce preparedness	Competency-based curriculum
4. Social, Political, and Economic Context of Public Health Diplomacy	Equity negotiations	Global South case studies
5. Conceptual development of Public Health Diplomacy	Defining shared responsibilities	Classroom discussions
6. Evaluation of Initiatives in Public Health Diplomacy	Monitoring treaty implementation	Evaluation framework assignments

### Consensus action items

We have several suggestions for advancing public health diplomacy goals based on the conclusions from the discussion session of the four working groups convened to discuss a series of broader goals for the advancement of public health diplomacy. Some of the initiatives are continuations of findings from the first Public Health Diplomacy summit (1). Each of the initiatives and their requisite action items can be found in Table 6.

**Table 6:** Initiatives and immediate action items.

Initiative	Action item	Relevant theme(s)
1. Create Public Health Diplomacy capacity-building group	1. Identify existing regional international public health skills, knowledge and competency frameworks and cross-cutting themes overlapping with diplomacy and other sectors critical to preparing a public health diplomat workforce.	‘Collaboration in Public Health Diplomacy’, ‘Professionalization of Public Health Diplomacy’, ‘Facilitation and Implementation of Initiatives’
2. Develop an online, interactive resource hub to disseminate Public Health Diplomacy research, Innovation, Skills and Experiential learning	2. Develop a centralized repository of case studies around the world reflecting public health diplomacy with lessons learned and opportunities ahead.	‘Conceptual Development of Public Health Diplomacy’, ‘Collaboration in Public Health Diplomacy’, ‘Evaluation of Initiatives in Public Health Diplomacy’.
3. Identify global and region-specific learning outcomes to prepare public health students to succeed in an interconnected and complex world through public health diplomacy	3. Develop a public health diplomacy curriculum among public health professionals to train a public health diplomat workforce.	‘Social, Political, and Economic Context of Public Health Diplomacy’, ‘Professionalization of Public Health Diplomacy’.
4. Organize events to help raise awareness about public health diplomacy in the field of public health	4. Launch a Public Health Diplomacy podcast bring local, regional, and international experts sharing examples of public health diplomacy and information up to date.	‘Collaboration in Public Health Diplomacy’, ‘Professionalization of Public Health Diplomacy’.
5. Develop a virtual community of Public Health Diplomats	5. Host a communication hub on a to-be-determined platform.	‘Collaboration in Public Health Diplomacy’, Conceptual Development of Public Health Diplomacy’, ‘Professionalization of Public Health Diplomacy’.

## Discussion

The findings from the Public Health Diplomacy 2nd Summit underscore the growing importance of public health diplomacy as a critical mechanism for addressing complex health challenges that transcend national boundaries. Across diverse regional perspectives from Europe, Africa, Latin America, and conflict-affected settings, participants consistently highlighted the need for stronger collaboration among governments, academic institutions, international organizations, civil society, and local communities. The discussions revealed that effective public health diplomacy extends beyond traditional diplomatic negotiations to include capacity building, leadership development, trust-building, knowledge translation, and community engagement. These findings demonstrate that strengthening public health systems and improving health security require integrated approaches that combine evidence-informed policymaking, cross-sectoral partnerships, and sustained investments in workforce development. Collectively, the summit emphasized that public health diplomacy has become an essential component of global health governance and a key strategy for promoting equity, resilience, and preparedness in an increasingly interconnected world.

The findings from this summit reinforce the growing recognition that public health diplomacy is an essential component of global health governance, particularly in addressing transnational health threats, health inequities, and fragile health systems. Participants consistently emphasized the importance of trust-building, multilateral collaboration, and local ownership. A contemporary example is the WHO Pandemic Agreement, which demonstrates how public health diplomacy facilitates consensus-building among countries on issues such as pathogen sharing, equitable access to medical countermeasures, and coordinated preparedness planning. The negotiations illustrate how sustained communication, trust, and multilateral collaboration are essential for addressing transnational public health threats (13). This also aligns with the framework proposed by Ilona Kickbusch (2007), who described global health diplomacy as a process that brings together public health, international affairs, and governance to improve population health outcomes while strengthening international relations (4). Similarly, this also supports findings by Kelley Lee and colleagues, who argued that effective global health diplomacy increasingly depends on non-state actors, academic institutions, and civil society networks working collaboratively across multiple governance levels (14).

The discussions demonstrated the critical role of workforce development, implementation science, and competency-based education in strengthening public health diplomacy capacities. Participants repeatedly noted the need to integrate leadership, and policy translation skills into public health curricula to prepare future professionals for increasingly complex global health challenges. These findings are reflected in the experiences shared by participants from Libya, where maintaining health services during prolonged conflict has required sustained coordination among government agencies, humanitarian organizations, healthcare providers, and local communities. Similar coordination mechanisms have been identified by WHO as essential for restoring health services and strengthening health system resilience in conflict-affected settings (15, 16). These findings are also consistent with previous literature emphasizing the need for interdisciplinary training in global health leadership and diplomacy (17, 18). These findings suggest that sustainable public health diplomacy requires not only institutional cooperation and political commitment, but also long-term investment in human capital, regional partnerships, and community-centered approaches.

Although public health diplomacy has gained increasing attention, important challenges remain. Differences in political priorities, economic resources, governance structures, and institutional capacities continue to influence how diplomacy is practiced across regions. Balancing national interests with global solidarity, ensuring equitable participation of low- and middle-income countries, and translating diplomatic agreements into measurable public health outcomes remain persistent challenges. These findings reinforce the need for stronger evaluation frameworks, competency development, and evidence-informed policy implementation.

The findings of this summit highlight the need to integrate public health diplomacy into public health education through competency-based curricula that emphasize leadership, negotiation, communication, and cross-sector collaboration. Schools and programs of public health can operationalize these competencies through case-based learning, policy simulations, experiential learning opportunities, and analyses of real-world examples. Aligning these competencies with existing accreditation frameworks, such as the Council on Education for Public Health (CEPH) competencies (19), would support the systematic preparation of future public health professionals to address increasingly complex global health challenges.

One of the major strengths of this summit paper is the inclusion of diverse perspectives from academic leaders, public health practitioners, policymakers, diplomats, and representatives of regional and international organizations across Europe, Africa, and Latin America. The discussions provided a unique opportunity to capture real-world experiences and practical examples of public health diplomacy across different contexts, including health system strengthening, conflict settings, pandemic preparedness, workforce development, and global health governance. The multidisciplinary and multi-regional nature of the summit enabled the identification of common themes and shared priorities, highlighting the growing importance of trust-building, collaboration, leadership, and capacity development in advancing public health diplomacy. Furthermore, the summit moved beyond theoretical discussions by presenting practical case studies and actionable recommendations that can inform future policy, education, and practice.

Several limitations should be acknowledged. The findings are based on presentations and discussions from summit participants and therefore reflect the perspectives and experiences of individuals and organizations represented at the event. As a result, the findings may not capture all regional perspectives or the full diversity of public health diplomacy practices globally. The qualitative nature of the discussions may introduce subjective interpretations, and the themes identified should not be considered exhaustive. Reliance on notetaker summaries alongside session transcripts may have introduced variability in interpretation and thematic synthesis despite efforts to ensure consistency in data extraction. In addition, English served as the primary language of discourse throughout the summit, which may have limited participation or nuanced expression from non-native English speakers. Because the summit was hosted at a U. S.-based academic institution, some discussions may also reflect institutional or contextual framing that could influence the prioritization and interpretation of public health diplomacy issues. Furthermore, while the summit included participants from multiple sectors and regions, the perspectives of some community-based organizations, private-sector stakeholders, and other non-state actors may have been underrepresented. Finally, the recommendations emerging from the summit have not yet been systematically evaluated for implementation or effectiveness and should be viewed as preliminary priorities for future action and research.

Therefore, future efforts should focus on validating these competencies through broader international stakeholder engagement, integrating them into accredited public health education programs, and evaluating their application using real-world policy initiatives.

## Conclusion

The discussions of the 2nd Public Health Diplomacy summit highlighted that public health diplomacy have considerably advanced the shared understanding about the critical need to strengthen global health security, equity, and resilience in an increasingly interconnected and politically complex world. The findings demonstrated that effective public health diplomacy depends not only on high-level multilateral negotiations, but also on trusted networks, community engagement, cross-sectoral collaboration, and sustained institutional partnerships that operate from the local to the global level. Participants emphasized the importance of capacity building, leadership development, implementation science, knowledge translation, and culturally responsive cooperation in public health.

The summit underscored the urgent need to integrate diplomacy competencies into public health education and workforce training including experiential learning opportunities, while promoting innovative financing, digital transformation, and regional self-reliance to reduce inequities and strengthen preparedness for future. The collective insights reinforced that public health diplomacy is not merely a policy concept, but a practical and action-oriented approach that builds trust, fosters solidarity, and enables sustainable solutions through collaboration, shared responsibility, and evidence-informed decision-making. The resultant consensus priority actions highlight a clear path forward not only for the field, but its integration into the greater public health landscape. Establishing public health diplomacy communities of practice, and global knowledge-sharing platforms may help translate summit recommendations into sustainable action and foster a more resilient and collaborative global public health ecosystem.
